# Antibiotic Resistance of Gram-Negative Bacteria in Rivers, United States

**DOI:** 10.3201/eid0807.010264

**Published:** 2002-07

**Authors:** Ronald J. Ash, Brena Mauck, Melissa Morgan

**Affiliations:** * Washburn University, Topeka, Kansas, USA

**Keywords:** antibiotics, antibiotic resistance, beta-lactam resistance, freshwater

## Abstract

Bacteria with intrinsic resistance to antibiotics are found in nature. Such organisms may acquire additional resistance genes from bacteria introduced into soil or water, and the resident bacteria may be the reservoir or source of widespread resistant organisms found in many environments. We isolated antibiotic-resistant bacteria in freshwater samples from 16 U.S. rivers at 22 sites and measured the prevalence of organisms resistant to β-lactam and non β-lactam antibiotics. Over 40% of the bacteria resistant to more than one antibiotic had at least one plasmid. Ampicillin resistance genes, as well as other resistance traits, were identified in 70% of the plasmids. The most common resistant organisms belonged to the following genera: *Acinetobacter*, *Alcaligenes*, *Citrobacter*, *Enterobacter*, *Pseudomonas*, and *Serratia*.

The presence of antibiotic-resistant bacteria in freshwater sources throughout the world has been documented (1–5). Selection of resistant organisms in nature may result from natural production of antibiotics by soil organisms, runoff from animal feed or crops, or waste products from treated animals or humans (6–10). Natural reservoirs of resistance genes may provide a source of transferable traits for emerging pathogens (6,11). The prevalence, nature, properties, and origin of such reservoirs in U.S. rivers have not been studied on a national scale. We frequently found organisms resistant to naturally occurring and human-modified antibiotics in U.S. rivers. A large proportion of the resistant organisms were found to contain plasmids with resistance traits.

## Methods

Sterile pipettes were used to collect triplicate 10-mL water samples at each site. Samples were collected in city limits at all locations; sites were usually 1 mile downstream of the downtown area. Each sample was collected at a depth of approximately 15 cm. Temperature and pH measurements were also made at the time of sampling. All samples, either undiluted or diluted in Luria-Bertani (LB) broth (12), were plated (0.1 mL, spread plate) on LB agar some with and some without ampicillin (150 µg/mL) at the collection site. Ampicillin was used to select for potential β-lactamase producers. Water samples were not stored or concentrated before plating. Plates were incubated at 30°C–32°C for 24 hours before the total number of bacteria and ampicillin-resistant bacteria were estimated. Variations in the number of CFU and ampicillin-resistant CFU did not differ (p=0.01) in a group of the triplicate samples plated at a particular time. Ampicillin-resistant isolates were picked to master LB plus ampicillin plates until further tested against additional antibiotics.

National Committee for Clinical Laboratory Standards (NCCLS) procedures and evaluation methods were used (13). Briefly, isolates were grown in LB plus ampicillin broth until the turbidity of a 0.5 MacFarland standard was reached. Cultures were swabbed on Mueller-Hinton agar, and antibiotic discs (Becton Dickinson and Co., Cockeysville, MD) were added. The antibiotics used were cefotaxime, ceftazidime, amoxicillin plus clavulanic acid, cephalothin, imipenem, kanamycin, streptomycin, chloramphenicol, tetracycline, and ciprofloxacin. MIC were determined with E test strips (AB Biodisk North America, Piscataway, NJ) under NCCLS conditions as described by the manufacturer. Isolates showing complete resistance to at least one antibiotic other than ampicillin were frozen in LB plus 10% dimethyl sulfoxide at -78°C and used as stocks for further testing. Freezing was preferred since isolate storage in the refrigerator for >2 weeks frequently resulted in loss of cultures. Nitrocefin discs (Cefinase, BD Microbiology Systems, Sparks, MD) were placed on bacterial cultures growing on Mueller Hinton agar and observed for the color change indicative of hydrolysis.

Organisms were grown overnight in LB with ampicillin broth with shaking. Two methods were used for every isolate examined: the boiling method (14) and alkaline lysis (15). Agarose gel electrophoresis in 0.7% agarose (GTG, FMC Corp., Rockland, ME) with 1X Tris/acetate/EDTA buffer (12) was used to determine the presence and size of plasmids. Plasmids were purified by removal of unstained gel slices and centrifugal elution through polyester fiberfill plugs (16).

The presence of resistance markers on purified plasmids was determined as follows: electrocompetent *Escherichia coli* (TOP 10 cells, Invitrogen, Inc., Carlsbad, CA) was mixed with 5 µL of pure plasmid DNA and subjected to 1.8 kV in a Bio-Rad Pulser (Bio-Rad Laboratories, Hercules, CA). Electroporated cells (cells with DNA physically introduced) were mixed with SOC medium (12) and incubated for 60 minutes at 37°C before plating on LB with ampicillin plates. Transformants were picked and checked for additional resistance traits as described above.

## Results

The number and range of ampicillin-resistant bacteria recovered at the 22 sites sampled in the past 3 years are shown in Table 1. Fourteen of the rivers were sampled more than once. Considerable variations in the total number of CFU and ampicillin-resistant organisms were encountered. The MIC for ampicillin was >256 for 98% of the ampicillin-resistant organisms tested. This high level of resistance, found in all rivers tested, was not unexpected since initial plating was on LB plates containing 150 µg/mL of the antibiotic. No apparent correlation existed between numbers of culturable or ampicillin-resistant organisms and temperature (range 0°C–28°C) or pH (range 7.2–8.7) in any of the rivers tested (data not shown). The number of resistant isolates growing on MacConkey agar was >90% for most samples. The ampicillin-resistant isolates were predominantly gram-negative nonlactose-fermenters (data not shown). Oxidase testing and biochemical characterization indicated that the major genera of these bacteria were *Acinetobacter, Alcaligenes, Citrobacter, Enterobacter, Pseudomonas,* and *Serratia*. *Klebsiella* and *Proteus* were also isolated but less frequently than the other organisms.

**Table 1:** Ampicillin-resistant bacteria, U.S. rivers

River	N^a^	log_10_ CFU/mL^b^	% ampicillin-resistant
Arkansas-Little Rock, AR	2	3.09–3.37 (3.25)	6.6–21.0
Arkansas-Witchita, KS	4	3.09–4.05 (3.66)	10.4–25.7
Canadian-Oklahoma City, OK	2	3.03–4.36 (4.08)	22.6–24.1
Chattahoochee-Atlanta, GA	2	2.32–2.66 (2.52)	34.5–38.4
Chicago-Chicago, IL	1	4.21	26.9
Colorado-Glenwood Springs, CO	1	2.56	59.2
Cuyahoga-Cleveland, OH	1	3.45	26.4
Des Moines-Des Moines, IA	3	2.66–3.81 (3.40)	13.7–34
Hudson-New York, NY	3	2.94–4.06 (3.80)	10.1–36.6
Kansas-Topeka, KS	24	2.86–4.15 (3.67)	4.9–52.5
Mississippi-New Orleans, LA	1	3.09	5.9
Mississippi-Minneapolis, MN	2	2.89–3.22 (3.09)	19.7–23.7
Mississippi-St. Louis, MO	4	3.93–4.61 (4.31)	6.7–73.0
Missouri-Parkville, MO	7	3.28–4.70 (4.08)	6.1–21.5
Ohio-Cincinnati, OH	6	2.86–4.80 (4.15)	20.0–53.0
Ohio-Louisville, KY	4	2.59–3.70 (3.22)	12.4–20.0
Ohio-Pittsburgh, PA	1	2.53	15.7
Ohio-Wheeling, WV	1	2.76	32.0
Platte-Grand Island, NE	3	3.20–3.56 (3.32)	3.5–33.9
Scioto-Columbus, OH	1	2.80	3.9
Wabash-Terre Haute, IN	3	2.81–3.19 (3.04)	17.0–25.0
White-Indianapolis, IN	1	4.36	22.5

^a^Refers to the number of visits to each site. ^b^Total CFU isolated on Luria-Bertani medium; range and mean (in parentheses) are given for sites sampled more than once.

The resistance of ampicillin-resistant isolates to other β-lactam antibiotics is presented in Table 2. Organisms resistant to cefotaxime, ceftazidime, and imipenem were detected at a number of sites. This finding prompted us to plate water samples on LB medium with cefotaxime (60 µg/mL). Cefotaxime -resistant bacteria were readily isolated from all rivers tested on this selective medium (Table 3). Of these cefotaxime-resistant organisms, 20% to 30% were gram-positive, spore-forming rods, which appeared to belong to the genus *Bacillus*. Although these gram-positive organisms may be important as reservoirs of resistance genes, only those cefotaxime-resistant isolates growing on MacConkey agar and shown to be gram negative were used for further analysis. Of the gram-negative cefotaxime-resistant organisms, 87% had a cefotaxime MIC >256. The remaining 13% had an MIC >48. Every cefotaxime-resistant isolate was capable of hydrolyzing nitrocefin, indicating the presence of β-lactamase. Many of the cefotaxime-resistant isolates were also resistant to ceftazidime (Table 4). Sixty-one per cent of the ceftazidime-resistant organisms (71 isolates tested) had a ceftazidime MIC >256, while 17% had an MIC of 12 to 192. Most (>80%) of the cefotaxime-resistant and ceftazidime-resistant organisms were identified as *Pseudomonas*.

**Table 2:** Resistance of ampicillin-resistant isolates to other β-lactam antibiotics, U.S. rivers^a^

		% resistant				
River	No. Tested	CEF	CTX	CAZ	IPM	AMC
Arkansas-Witchita, KS	43	—^a^	5	2	5	—
Canadian-Oklahoma City, OK	50	—	6	0	0	—
Chattahoochee-Atlanta, GA	70	93	13	—	—	—
Chicago-Chicago, IL	81	96	9	0	0	—
Colorado-Glenwood Springs, CO	87	94	25	1	10	—
Cuyahoga-Cleveland, OH	79	91	12	6	0	68
Des Moines-Des Moines, IA	63	8	0	0	0	8
Hudson-New York, NY	107	86	9	0	—	—
Kansas-Topeka, KS	199	25	20	8	4	—
Missouri-Parkville, MO	203	73	21	5	1	—
Ohio-Louisville, KY	85	67	4	2	0	10
Ohio-Cincinnati, OH	91	77	0	0	0	37
Ohio-Pittsburgh, PA	54	78	8	9	—	36
Ohio-Wheeling, WV	50	36	0	0	6	0
Platte-Grand Island, NE	245	96	16	2	—	24
Scioto-Columbus, OH	67	86	2	0	—	33
Mississippi-Minneapolis, MN	72	38	0	0	0	38
Mississippi-St. Louis, MO	51	98	4	0	—	57
Wabash-Terre Haute, IN	81	94	7	0	0	—
White-Indianapolis, IN	83	96	39	—	—	—

^a^Abbreviations used: —,not tested; CEF,cephalothin; CTX, cefotaxime; CAZ, ceftazidime; IPM, imipenem; AMC, amoxicillin+clavalanic acid.

**Table 3:** Isolation of cefotaxime-resistant bacteria, U.S. rivers

	log CFU/mL (%)		
River	LB^a^	LB+ampicillin	LB+ cefotaxime
Arkansas-Witchita, KS	3.24	2.65 (25.7)^a^	1.50 (1.8)
Canadian-Oklahoma City, OK	4.36	3.74 (24.1)	2.72 (2.3)
Chicago-Chicago, IL	4.21	3.64 (26.9)	2.37 (1.4)
Des Moines-Des Moines, IA	3.81	2.95 (13.7)	2.09 (1.9)
Hudson-New York, NY	2.94	2.46 (32.8)	1.65 (5.1)
Kansas-Topeka, KS	4.49	4.21 (52.5)	3.23 (5.5)
Mississippi-St. Louis, MO	3.99	3.29 (20.1)	2.51 (3.3)
Missouri-Parkville, MO	4.70	3.96 (18.2)	3.29 (3.9)
Ohio-Louisville, KY	2.70	1.86 (14.4)	0.60 (0.8)
Ohio-Cincinnati, OH	2.89	2.20 (20.0)	0.48 (0.3)
Platte-Grand Island, NE	4.23	3.45 (16.7)	2.74 (3.2)
Wabash-Terre Haute, IN	3.19	2.59 (25.0)	1.75 (3.7)

^a^ LB, Luria-Bertani broth.

**Table 4:** Cefotaxime-resistant isolates, U.S. rivers

River	Cefotaxime-resistant/total tested (%)
Canadian-Oklahoma City, OK	16/50 (32.0)
Chicago-Chicago, IL	14/32 (43.7)
Des Moines-Des Moines, IA	19/28 (67.8)
Kansas-Topeka, KS	15/28 (53.5)
Mississippi-St. Louis, MO	41/43 (95.3)
Missouri-Parkville, MO	28/49 (57.1)
Platte-Grand Island, NE	12/73 (16.4)

The resistance of ampicillin-resistant isolates to non-β–lactam antibiotics is shown in Table 5. Many organisms in the rivers had resistance to at least one antibiotic other than ampicillin, and a substantial fraction were able to survive a number of antibiotics. One gram-negative organism resistant to ciprofloxacin was found in the 3,011 ampicillin-resistant isolates tested. Gram-positive ciprofloxacin-resistant bacteria were more numerous and were readily isolated.

**Table 5:** Resistance of ampicillin-resistant isolates to non-β–lactam antibiotics^a^

		Ampicillin-resistant isolates	
River	No. tested	Ampicillin + 1 (%)	Ampicillin >1 (%)
Arkansas-Little Rock, AR	80	3 (3.7)^a^	1 (1.2)
Arkansas-Witchita, KS	155	31 (20)	3 (1.9)
Canadian-Oklahoma City, OK	42	6 (14)	2 (4.7)
Chattahoochee-Atlanta, GA	101	21 (20.8)	2 (2.0)
Chicago-Chicago, IL	100	43 (43)	12 (12)
Colorado-Glenwood Springs, CO	100	32 (32)	24 (24)
Cuyahoga-Cleveland, OH	79	28 (35.4)	10 (12.6)
Des Moines-Des Moines, IA	105	50 (47.6)	8 (7.6)
Hudson-New York, NY	108	20 (18.5)	7 (6.5)
Kansas-Topeka, KS	104	44 (42.3)	2 (1.9)
Mississippi-New Orleans, LA	42	10 (23.8)	4 (9.5)
Mississippi-Minneapolis, MN	115	19 (16.5)	7 (6.0)
Mississippi-St. Louis, MO	161	46 (28.8)	10 (6.2)
Missouri-Parkville, MO	182	30 (16.4)	11 (6.0)
Ohio-Cincinnati, OH	144	16 (11.1)	4 (2.7)
Ohio-Louisville, KY	141	22 (15.6)	7 (4.9)
Ohio-Pittsburgh, PA	54	14 (25.9)	5 (9.2)
Ohio-Wheeling, WV	50	2 (4)	0 (0)
Platte-Grand Island, NE	65	11 (16.9)	3 (4.6)
Scioto-Columbus, OH	59	10 (16.9)	3 (5.0)
Wabash-Terre Haute, IN	109	30 (27.5)	12 (11)
White-Indianapolis, IN	106	17 (16)	5 (4.7)

^a^ Ampicillin + 1 = resistance to ampicillin and at least one non-β–lactam. Ampicillin >1 = resistance to ampicillin and 2 or more non-β–lactams. Non-β–lactam antibiotics tested: ciprofloxacin, tetracycline, chloramphenicol, kanamycin, and streptomycin.

Two different plasmid isolation procedures were used to analyze the isolates resistant to ampicillin and one other antibiotic. Of the 374 isolates, 167 (44%) contained at least one plasmid. This number probably represents a minimal estimate since the methods used may not be optimal for all species encountered. Plasmids ranged in size from 2 kb to >23 kb. Purified plasmids were checked for their ability to transform *E. coli*. Of the 54 plasmids tested, 38 (70%) contained the gene for ampicillin resistance. Further, 97% of the plasmids with the ampicillin-resistant gene also carried at least one other resistance trait.

## Discussion

We found that culturable antibiotic-resistant bacteria were widespread in nonconcentrated water samples from many U.S. rivers. This finding, in and of itself, is not surprising since the intrinsic resistance of many organisms to antibiotics is well documented (17). Bacteria that are resistant to chemically modified and synthesized antibiotics are also widespread in the environment. The use of selective media resulted in the isolation of gram-negative organisms with high levels of resistance (MIC >256 µg) to the clinically useful β-lactams ampicillin, cefotaxime, and ceftazidime. Organisms resistant to imipenem were also frequently isolated. Nitrocefin hydrolysis data suggest that β-lactamase production is a major mechanism of resistance to ampicillin in river isolates. The resistance of natural isolates to ceftazidime and cefotaxime strongly suggests that these organisms may produce extended-spectrum β-lactamases (ESBL), since resistance to these third-generation cephalosporins is considered the single most important indicator of ESBL (18,19). Alternatively, chromosomal AmpC β-lactamases may be responsible for the resistance of *Pseudomonas* to cefotaxine and ceftazidime (19). Distinguishing between these possibilities will be important in determining transmissibility of β-lactamase resistance.

Many of the organisms resistant to ampicillin and at least one other antibiotic (40%) harbored plasmids. Although two methods were used to isolate plasmids, some bacteria may have been refractory to these procedures or a large or low copy number plasmids were not observed. Resistance to ampicillin and other antibiotics was plasmid-borne, as indicated by electroporation of cells with purified plasmids.

The results presented here have limitations and must be considered in light of the fact that many aquatic organisms are probably nonculturable (20). The bacteria that cannot be cultivated may be part of the reservoir of resistance genes as well. PCR has been used to identify nonculturable bacteria in stream sediments (21). This technique may be used to identify antibiotic resistance genes in nonculturable organisms as well.

Many of the ampicillin-resistant isolates were also resistant to non-β–lactam antibiotics (Table 5). The frequency with which these organisms were found suggests that characterization of resistance genes and the plasmids on which they reside should provide information about reservoirs for antibiotic resistance in the environment.
